# Supplementary material for: Evolution of Inflammatory and Oxidative Stress Markers in Romanian Obese Male Patients with Type 2 Diabetes Mellitus after Laparoscopic Sleeve Gastrectomy: One Year Follow-Up
Source: Metabolites. 2020 Jul 28;10(8):308. doi: 10.3390/metabo10080308 (PMC7464585; doi:10.3390/metabo10080308)
Supplement: Supplementary file 1 [file metabolites-10-00308-s001.pdf]

Evolution of Inflammatory and Oxidative Stress Markers in Romanian Obese Male Patients with Type 2 Diabetes Mellitus after Laparoscopic Sleeve Gastrectomy: One Year Follow-Up

Ariana Picu [1,2,†], Laura Petcu [1,2,†,*], Diana Simona Ştefan [1], Grațiela Grădișteanu Pîrcălăbioru [2,3,*], Manuela Mitu [1], Daiana Bajko [1], Daniela Lixandru [4], Cristian Guja [1,5], Octavian Savu [1,6], Anca Pantea Stoian [5], Alina Constantin [7], Bogdan Smeu [8], Cătălin Copăescu [8], Mariana Carmen Chifiriuc [2,3,*], Elena Ionica [2] and Constantin Ionescu-Tîrgovişte [1,9]

Supplemental Table 1. Results of Clinical, Biochemical, Anthropometrical and Metabolic Status Parameters Determination in the Two Studied Groups at Baseline (V1).

Clinical and Biochemical Parameters	CTG	LSG	*P* value
	n=21	n=20	
Waist Circumference (WC) (cm)	135.9±10.72	139.85±16.69	0.37
BMI (kg/m^2)	41.51±5.56	41.2±4.8	0.85
Systolic Blood Pressure (SBP) (mmHg)	135.29±13.59	131.58±16.33	0.43
Diastolic Blood Pressure (DBP) (mmHg)	83.8±8.08	76.05±11.37	0.17
HbA1c (%)	8.35±1.49	8.82±1.56	0.33
Glycaemia (mg/dl)	213.19±84.17	220.9±90.33	0.77
Insulin (µUI/mL)	28.41±3.76	23.71±3.74	0.38
HOMA-IR (%)	14.48±5.47	14.88±5.88	0.82
HOMA-β (%)	129.77±63.07	132.79±85.76	0.89
Proinsulin (pmol/L)	11.72±2.25	7.67±1.86	0.21
Proinsulin/Insulin	0.46±0.09	0.37±0.06	0.46
C Peptide (ng/ml)	9.40±0.47	9.36±0.46	0.95
Total cholesterol (mg/dl)	203.2±33.41	182.49±36.40	0.06
HDL-cholesterol (mg/dl)	37.19±6.36	34.62±13.67	0.44
Triglycerides (mg/dl)	211.10±92.51	218.94±117.77	0.81
LDL-cholesterol (mg/dl)	123.79±33.8	104.08±39.70	0.09
Uric Acid (mg/dl)	5.65±2.05	5.99±1.84	0.58
Creatinine (mg/dl)	1.10±0.24	1.13±0.20	0.77
Urea (mg/dl)	39.74±20.82	40.55±7.58	0.87
AST	36.02±4.45	35.89±4.82	0.98
ALT	65.03±15.24	47.43±6.19	0.3
GGT	106.42±38.16	59.52±8.15	0.24
Albumin (g/dl)	4.55±0.39	4.44±0.32	0.35
Total Protein (g/dl)	7.22±0.54	7.1±0.59	0.49
RMR measured (kcal/day)	2381.2 ± 462.15	2512.7 ± 440.25	0.36
RMR predicted (kcal/day)	2438.5 ± 285.7	2413.05 ± 254.98	0.76
VO_2 in rest state (ml/min)	347.6 ± 68.19	364.4 ± 62.82	0.42

**VCO_2 in rest state (ml/min)**	272.85 ± 52.64	295.35 ± 57.19	0.20
**Fat Mass (%)**	35 ± 5.32	31.12 ± 8.74	0.09
**Free Fat Mass (kg)**	81.9 ± 13.85	87.92 ± 14.39	0.18

Mean ± SD;.

Supplemental Table 2. Comparison of clinical and metabolic characteristics determined at V1 and V2 for the two study groups.

**Clinical and Biochemical Parameters**	**CTG**		**p**	**LSG**		**p**
	**V1**	**V2**		**V1**	**V2**	
	**n=21**	**n=17**		**n=20**	**n=19**	
**Waist Circumference (cm)***	**139.0 (14.0)**	133.00 (10.00)	**0.001 [1]**	134.0 (16.0)	105.0 (10.5)	**<0.001[1]**
**BMI (kg/m^2)***	40.50 (8.60)	39.10 (8.30)	**<0.001[1]**	39.60 (4.90)	29.30 (2.25)	**<0.001[1]**
**SBP (mmHg)***	135.0 (12.0)	120.00 (10.00)	**<0.05[1]**	130.00(13.75)	125.0 (30.50)	0.3256 [1]
**DBP (mmHg)***	90.00 (10.00)	80.00 (20.00)	0.262 [1]	80.00 (10.00)	82.00 (13.00)	**<0.05[1]**
**HbA1c (%)***	7.56 (1.87)	7.91 (2.62)	0.5791 [1]	8.40 (1.45)	6.60 (0.75)	**<0.001[1]**
**Glycaemia (mg/dl)***	184.5 (99.3)	145.17 (103.63)	0.2842 [1]	197.7 (107.4)	99.56 (27.48)	**<0.001[1]**
**Total cholesterol (mg/dl)#**	194.14±26.75	191.16±55.01	0.08 [2]	184.65±36.04	184.55±32.05	0.99 [2]
**HDL-cholesterol (mg/dl)***	36.10 (8.20)	35.30 (9.50)	0.2446 [1]	34.20 (8.15)	39.20 (9.90)	**<0.05[1]**
**Triglycerides (mg/dl)***	187.4 (123.26)	167.35 (100.68)	0.5791 [1]	178.23 (137.50)	89.43 (48.30)	**<0.001[1]**
**LDL-cholesterol (mg/dl)***	121.15 (31.6)	119.86 (42.86)	0.2247 [1]	111.15 (34.7)	126.54 (17.5)	**0.06 [1]**
**Uric Acid (mg/dl)***	5.99 (1.45)	6.38 (1.54)	0.1594 [1]	5.89 (2.34)	6.51 (2.19)	0.2753 [1]
**Creatinine (mg/dl)***	1.02 (0.10)	1.10 (0.22)	0.9058 [1]	1.09 (0.19)	1.00 (0.15)	**<0.05[1]**
**Urea (mg/dl)***	35.25 (7.12)	33.92 (8.19)	0.7119 [1]	40.18 (9.31)	36.93 (11.07)	0.8596 [1]
**AST (IU/L)***	24.34 (12.45)	20.51 (14.75)	**0.07 [1]**	29.60 (23.58)	16.74 (3.68)	**<0.001[1]**
**ALT (IU/L)***	36.18 (17.38)	33.06 (24.66)	0.1743 [1]	41.38 (46.41)	15.36 (6.16)	**<0.001[1]**
**GGT (IU/L)***	45.34 (24.00)	33.46 (13.15)	**0.07 [1]**	53.91 (40.11)	28.57 (22.34)	**<0.05[1]**
**Albumin (g/dl)***	4.59 (0.43)	4.55 (0.28)	0.3087 [1]	4.48 (0.32)	4.58 (0.23)	0.4444 [1]
**Total Protein (g/dl)***	7.28 (0.74)	7.33 (0.45)	0.246 [1]	7.27 (0.82)	7.08 (0.65)	0.7475

1

* - Median and IQR range; # - mean ± SD; 1 - Wilcoxon signed rank test; 2 - Paired T Test.

Table 3. Comparison of clinical and metabolic characteristics determined at V1 and V3 for the two study groups.

Clinical and Biochemical Parameters	CTG			LSG		
	V1 n=21	V3 n=15	P value V1 vs. V3	V1 n=20	V3 n=19	P value V1 vs. V3
**Waist Circumference (cm)***	**139.0 (14.0)**	128.00 (13.00)	<0.001[1]	134.0 (16.0)	102.0 (10.25)	<0.001[1]
**BMI (kg/m^2)***	40.50 (8.60)	40.30 (7.50)	<0.05[1]	39.60 (4.90)	28.70 (1.50)	<0.001[1]
**SBP (mmHg)***	135.0 (12.0)	130.00 (19.00)	0.8504 [1]	130.00(13.75)	128.0 (31.50)	1.000[1]
**DBP (mmHg)***	90.00 (10.00)	92.00 (11.00)	0.1236 [1]	80.00 (10.00)	86.00 (22.00)	<0.05[1]
**HbA1c (%)***	7.56 (1.87)	8.74 (2.69)	0.804 [1]	8.40 (1.45)	5.90 (0.69)	<0.001[1]
**Glycaemia (mg/dl)***	190.1 (168.0)	163.80 (119.97)	0.1354 [1]	197.7 (107.4)	96.33 (29.98)	<0.001[1]
**Total cholesterol (mg/dl)#**	208.91±37.66	196.06 (±44.23)	<0.05[2]	184.65±36.04	188.75±41.84	0.7105 [2]
**HDL-cholesterol (mg/dl)***	35.70 (9.60)	32.40 (15.45)	0.9547 [1]	34.20 (8.15)	50.70 (15.65)	<0.001[1]
**Triglycerides (mg/dl)***	201.10 (116.85)	147.62 (111.41)	0.05[1]	178.23 (137.50)	86.26 (64.14)	<0.001[1]
**LDL-cholesterol (mg/dl)***	121.15 (31.6)	130.43 (50.94)	0.804 [1]	111.15 (34.7)	111.4 (61.53)	0.2935 [1]
**Uric Acid (mg/dl)***	5.99 (1.45)	5.96 (1.92)	0.7197 [1]	5.89 (2.34)	5.59 (1.34)	0.9217 [1]
**Creatinine (mg/dl)***	1.02 (0.10)	1.04 (0.22)	0.164 [1]	1.09 (0.19)	1.02 (0.12)	0.06409 [1]
**Urea (mg/dl)***	35.25 (7.12)	37.84 (11.62)	0.5995 [1]	40.18 (9.31)	49.88 (14.72)	<0.001[1]
**AST (IU/L)***	24.34 (12.45)	22.10 (9.53)	0.2293 [1]	29.60 (23.58)	17.38 (5.32)	<0.001[1]
**ALT (IU/L)***	36.18 (17.38)	30.52 (12.71)	0.2293 [1]	41.38 (46.41)	22.80 (9.63)	<0.05[1]
**GGT (IU/L)***	45.34 (24.00)	33.93 (8.83)	0.1876 [1]	53.91 (40.11)	30.95 (20.36)	<0.05[1]
**Albumin (g/dl)***	4.59 (0.43)	4.62 (0.31)	0.3626 [1]	4.48 (0.32)	4.54 (0.30)	0.7022 [1]

**Total Protein (g/dl)***	7.28 (0.74)	7.29 (0.55)	0.4212 [1]	7.27 (0.82)	7.11 (0.74)	0.5066 [1]
**RMR measured (kcal/day)***	2218 (557.50)	2073 (274.00)	0.8926 [1]	2489 (471)	1732 (276)	**<0.001[1]**
**RMR predicted (kcal/day)***	2413.43 ±276.73	2372.84 (±286.59)	**<0.05[2]**	2392.31±244.03	1846.31±175.58	**<0.001[2]**
**RMRm - RMRp**	-118.68 ±412.26	-150.38 ±356.41	0.6981 [2]	119.89±396.91	-125.00±170.52	**<0.05[2]**
**Fat Mass (%)***	36.50 (7.80)	34.80 (7.65)	**<0.05[1]**	32.70 (9.85)	20.30 (7.60)	**<0.001[1]**
**Free Fat Mass (kg)***	77.40 (10.50)	81.10 (10.25)	**<0.05[1]**	86.96 (20.50)	71.50 (9.60)	**<0.001[1]**
**Visceral Fat Level (%)#**	20,86±5	19,2±4,92	0.331	21,65±5,28	10±4,07	**<0.001**

* - Median and IQR range; # - mean ± SD; 1 - Wilcoxon signed rank test; 2 - Paired T Test.
